# Clinical correlates and prognostic impact of neurologic disorders in Takotsubo syndrome

**DOI:** 10.1038/s41598-021-01496-9

**Published:** 2021-12-07

**Authors:** Victoria L. Cammann, Jan F. Scheitz, Regina von Rennenberg, Lutz Jäncke, Christian H. Nolte, Konrad A. Szawan, Helena Stengl, Michael Würdinger, Matthias Endres, Christian Templin, Jelena R. Ghadri, InterTAK Consortium, Rodolfo Citro, Carmine Vecchione, Eduardo Bossone, Sebastiano Gili, Michael Neuhaus, Jennifer Franke, Benjamin Meder, Miłosz Jaguszewski, Michel Noutsias, Maike Knorr, Thomas Jansen, Fabrizio D’Ascenzo, Wolfgang Dichtl, Christof Burgdorf, Behrouz Kherad, Carsten Tschöpe, Annahita Sarcon, Jerold Shinbane, Lawrence Rajan, Guido Michels, Roman Pfister, Alessandro Cuneo, Claudius Jacobshagen, Mahir Karakas, Wolfgang Koenig, Alexander Pott, Philippe Meyer, Marco Roffi, Adrian Banning, Mathias Wolfrum, Florim Cuculi, Richard Kobza, Thomas A. Fischer, Tuija Vasankari, K. E. Juhani Airaksinen, L. Christian Napp, Rafal Dworakowski, Philip MacCarthy, Christoph Kaiser, Stefan Osswald, Leonarda Galiuto, Christina Chan, Paul Bridgman, Daniel Beug, Clément Delmas, Olivier Lairez, Ekaterina Gilyarova, Alexandra Shilova, Mikhail Gilyarov, Ibrahim El-Battrawy, Ibrahim Akin, Karolina Poledniková, Petr Toušek, David E. Winchester, Jan Galuszka, Christian Ukena, Gregor Poglajen, Pedro Carrilho-Ferreira, Christian Hauck, Carla Paolini, Claudio Bilato, Yoshio Kobayashi, Ken Kato, Toshihiro Shoji, Iwao Ishibashi, Masayuki Takahara, Toshiharu Himi, Jehangir Din, Ali Al-Shammari, Abhiram Prasad, Charanjit S. Rihal, Kan Liu, P. Christian Schulze, Matteo Bianco, Lucas Jörg, Hans Rickli, Gonçalo Pestana, Thanh H. Nguyen, Michael Böhm, Lars S. Maier, Fausto J. Pinto, Petr Widimský, Stephan B. Felix, Ruediger C. Braun-Dullaeus, Wolfgang Rottbauer, Gerd Hasenfuß, Burkert M. Pieske, Heribert Schunkert, Monika Budnik, Grzegorz Opolski, Martin Borggrefe, Holger Thiele, Johann Bauersachs, Hugo A. Katus, John D. Horowitz, Carlo Di Mario, Thomas Münzel, Filippo Crea, Jeroen J. Bax, Frank Scherff, David Niederseer, Thomas F. Lüscher

**Affiliations:** 1 Department of Cardiology, University Heart Center, University Hospital Zurich, Raemistrasse 100, 8091 Zurich, Switzerland; 2 Center for Stroke Research Berlin and Department of Neurology With Experimental Neurology, Charité-Universitätsmedizin Berlin, Berlin, Germany; 3 DZHK (German Centre for Cardiovascular Research), Partner Site Berlin, Berlin, Germany; 4 Berlin Institute of Health (BIH), Berlin, Germany; 5 Division Neuropsychology, Department of Psychology, University of Zurich, Zurich, Switzerland; 6 DZNE (German Center for Neurodegenerative Disease), Partner Site Berlin, Berlin, Germany; 7 Heart Department, University Hospital “San Giovanni di Dio e Ruggi d’Aragona”, Salerno, Italy; 8 Division of Cardiology, “Antonio Cardarelli” Hospital, Naples, Italy; 9 Centro Cardiologico Monzino, IRCCS, Milan, Italy; 10 Department of Cardiology, Kantonsspital Frauenfeld, Frauenfeld, Switzerland; 11 Department of Cardiology, Heidelberg University Hospital, Heidelberg, Germany; 12 First Department of Cardiology, Medical University of Gdansk, Gdańsk, Poland; 13 Division of Cardiology, Angiology and Intensive Medical Care, Department of Internal Medicine III, Mid-German Heart Center, University Hospital Halle, Martin-Luther-University Halle-Wittenberg, Halle (Saale), Germany; 14 Center for Cardiology, Cardiology 1, University Medical Center Mainz, Mainz, Germany; 15 Division of Cardiology, Department of Medical Sciences, AOU Città della Salute e della Scienza, University of Turin, Turin, Italy; 16 University Hospital for Internal Medicine III (Cardiology and Angiology), Medical University Innsbruck, Innsbruck, Austria; 17 Heart and Vascular Centre Bad Bevensen, Bad Bevensen, Germany; 18 Department of Cardiology, Charité, Campus Rudolf Virchow, Berlin, Germany; 19 Section of Cardiac Electrophysiology, Department of Medicine, University of California-San Francisco, San Francisco, CA USA; 20 Keck School of Medicine, University of Southern California, Los Angeles, CA USA; 21 TJ Health Partners Heart and Vascular, Glasgow, KY USA; 22 Klinik für Akut- und Notfallmedizin, St.-Antonius-Hospital gGmbH, Akademisches Lehrkrankenhaus der RWTH Aachen, Eschweiler, Germany; 23 Department of Internal Medicine III, Heart Center University of Cologne, Cologne, Germany; 24 Krankenhaus “Maria Hilf” Medizinische Klinik, Stadtlohn, Germany; 25 Clinic for Cardiology and Pneumology, Georg August University Goettingen, Goettingen, Germany; 26 Department of General and Interventional Cardiology, University Heart Center Hamburg, Hamburg, Germany; 27 DZHK (German Centre for Cardiovascular Research), Partner Site Hamburg/Kiel/Luebeck, Hamburg, Germany; 28 Deutsches Herzzentrum München, Technische Universität München, Munich, Germany; 29 DZHK (German Centre for Cardiovascular Research), Partner Site Munich Heart Alliance, Munich, Germany; 30 Department of Internal Medicine II – Cardiology, Medical Center, University of Ulm, Ulm, Germany; 31 Service de Cardiologie, Hôpitaux Universitaires de Genève, Geneva, Switzerland; 32 Department of Cardiology, John Radcliffe Hospital, Oxford University Hospitals, Oxford, UK; 33 Department of Cardiology, Kantonsspital Lucerne, Lucerne, Switzerland; 34 Department of Cardiology, Kantonsspital Winterthur, Winterthur, Switzerland; 35 Heart Center, Turku University Hospital and University of Turku, Turku, Finland; 36 Department of Cardiology and Angiology, Hannover Medical School, Hannover, Germany; 37 Department of Cardiology, King’s College Hospital, London, UK; 38 Department of Cardiology, University Hospital Basel, Basel, Switzerland; 39 Fondazione Policlinico Universitario A. Gemelli IRCCS, Università Cattolica del Sacro Cuore, Rome, Italy; 40 Department of Cardiology, Christchurch Hospital, Christchurch, New Zealand; 41 Department of Cardiology and Internal Medicine B, University Medicine Greifswald, Greifswald, Germany; 42 DZHK (German Centre for Cardiovascular Research), Partner Site Greifswald, Greifswald, Germany; 43 Department of Cardiology and Cardiac Imaging Center, University Hospital of Rangueil, Toulouse, France; 44 Intensive Coronary Care Unit, Moscow City Hospital # 1 Named After N. Pirogov, Moscow, Russia; 45 First Department of Medicine, Faculty of Medicine, University Medical Centre Mannheim (UMM) University of Heidelberg, Mannheim, Germany; 46 DZHK (German Center for Cardiovascular Research), Partner Site, Heidelberg-Mannheim, Mannheim, Germany; 47 Cardiocenter, Third Faculty of Medicine, Charles University in Prague and University Hospital Královské Vinohrady, Prague, Czech Republic; 48 Division of Cardiovascular Medicine, Department of Medicine, College of Medicine, University of Florida, Gainesville, FL USA; 49 Department of Internal Medicine I - Cardiology, University Hospital Olomouc, Olomouc, Czech Republic; 50 Klinik für Innere Medizin III, Universitätsklinikum des Saarlandes, Homburg, Saar Germany; 51 Advanced Heart Failure and Transplantation Center, University Medical Center Ljubljana, Ljubljana, Slovenia; 52 CHULN, Center of Cardiology of the University of Lisbon, Lisbon School of Medicine, Lisbon Academic Medical Center, Santa Maria University Hospital, Lisbon, Portugal; 53 Klinik und Poliklinik für Innere Medizin II, Universitätsklinikum Regensburg, Regensburg, Germany; 54 Local Health Unit n.8, Cardiology Unit, Arzignano, Vicenza, Italy; 55 Department of Cardiovascular Medicine, Chiba University Graduate School of Medicine, Chiba, Japan; 56 Department of Cardiology, Chiba Emergency Medical Center, Chiba, Japan; 57 Division of Cardiology, Kimitsu Central Hospital, Kisarazu, Japan; 58 Dorset Heart Centre, Royal Bournemouth Hospital, Bournemouth, UK; 59 Department of Cardiovascular Diseases, Mayo Clinic, Rochester, MN USA; 60 Division of Cardiology, Heart and Vascular Center, University of Iowa, Iowa City, IA USA; 61 Department of Internal Medicine I, University Hospital Jena, Friedrich-Schiller-University Jena, Jena, Germany; 62 Division of Cardiology, A.O.U San Luigi Gonzaga, Orbassano, Turin, Italy; 63 Department of Cardiology, Kantonsspital St. Gallen, St. Gallen, Switzerland; 64 Department of Cardiology, E.P.E, Centro Hospitalar Universitário de São João, Porto, Portugal; 65 Department of Cardiology, Basil Hetzel Institute, Queen Elizabeth Hospital, University of Adelaide, Adelaide, Australia; 66 Department of Internal Medicine, Cardiology and Angiology, Magdeburg University, Magdeburg, Germany; 67 Department of Cardiology, Medical University of Warsaw, Warsaw, Poland; 68 Department of Internal Medicine/Cardiology, Heart Center Leipzig – University Hospital, Leipzig, Germany; 69 Structural Interventional Cardiology, Careggi University Hospital, Florence, Italy; 70 Department of Cardiology, Leiden University Medical Centre, Leiden, The Netherlands; 71 Center for Molecular Cardiology, Schlieren Campus, University of Zurich, Zurich, Switzerland; 72 Royal Brompton and Harefield Hospitals Trust and Imperial College, London, UK

**Keywords:** Cardiology, Outcomes research

## Abstract

Cardiac alterations are frequently observed after acute neurological disorders. Takotsubo syndrome (TTS) represents an acute heart failure syndrome and is increasingly recognized as part of the spectrum of cardiac complications observed after neurological disorders. A systematic investigation of TTS patients with neurological disorders has not been conducted yet. The aim of the study was to expand insights regarding neurological disease entities triggering TTS and to investigate the clinical profile and outcomes of TTS patients after primary neurological disorders. The International Takotsubo Registry is an observational multicenter collaborative effort of 45 centers in 14 countries (ClinicalTrials.gov, identifier NCT01947621). All patients in the registry fulfilled International Takotsubo Diagnostic Criteria. For the present study, patients were included if complete information on acute neurological disorders were available. 2402 patients in whom complete information on acute neurological status were available were analyzed. In 161 patients (6.7%) an acute neurological disorder was identified as the preceding triggering factor. The most common neurological disorders were seizures, intracranial hemorrhage, and ischemic stroke. Time from neurological symptoms to TTS diagnosis was ≤ 2 days in 87.3% of cases. TTS patients with neurological disorders were younger, had a lower female predominance, fewer cardiac symptoms, lower left ventricular ejection fraction, and higher levels of cardiac biomarkers. TTS patients with neurological disorders had a 3.2-fold increased odds of in-hospital mortality compared to TTS patients without neurological disorders. In this large-scale study, 1 out of 15 TTS patients had an acute neurological condition as the underlying triggering factor. Our data emphasize that a wide spectrum of neurological diseases ranging from benign to life-threatening encompass TTS. The high rates of adverse events highlight the need for clinical awareness.

## Introduction

Patients with acute neurological disorders are susceptible to experience cardiac complications such as acute myocardial infarction, heart failure, arrhythmias, or cardiac arrest^1–6^. Takotsubo syndrome (TTS) is an emerging cardiovascular condition and represents an acute heart failure syndrome, which prototypically affects elderly women after a preceding triggering event^7^. Due to left ventricular recovery within weeks after the acute event it was widely anticipated that TTS is a self-limiting and rather benign cardiac condition. However, recent studies demonstrated that TTS is accompanied by similar mortality rates as acute myocardial infarction^8,9^. The underlying pathophysiological mechanisms of TTS have not been elucidated yet, but there is convincing evidence from brain imaging studies that altered physiological function within the brain heart axis might play a pivotal role^10,11^. A cross-sectional study based on national inpatient sample data has shown that 0.06% of hospitalization for primary acute neurological disorders are complicated by TTS and that incidence rates of TTS diagnosis among patients with acute neurological disorders have gradually increased during the entire study period^12^. Furthermore, the overall prevalence of acute neurological disorders is reported to be nearly 10 times higher in patients with TTS than in age- and sex-matched controls with acute myocardial infarction^8^.

Over the last years, uncontrolled case reports and single-center studies have added numerous acute neurological disorders as potential triggering factors of TTS^13–15^. However, a systematic characterization of neurological disorders triggering TTS is lacking. Therefore, we have designed the present study to extensively investigate frequency and spectrum of neurological disorders, clinical characteristics, and outcomes of patients with TTS and primary neurological disorders from the International Takotsubo Registry.

## Methods

### Study population

Patients were enrolled from the International Takotsubo Registry (InterTAK Registry, www.takotsubo-registry.com) which is a multicenter observational study including data from 45 sites in 14 countries (Australia, Austria, Czech Republic, Finland, France, Germany, Italy, New Zealand, Poland, Portugal, Russia, Switzerland, United Kingdom and United States). The rationale and methodological structure of the InterTAK Registry have been detailed previously^8,16^. Patients were included in the registry if International Diagnostic Takotsubo Criteria were met^7^. For the present study, the InterTAK Registry was screened for patients who had complete documentation on acute neurological disorders preceding the TTS event. Cases with acute neurological disorders were reviewed independently by two experienced board-certified neurologists and patients were categorized as Neuro-TTS if the following criteria were fulfilled: (i) evidence of a clearly definable disease entity; (ii) first symptoms of the acute neurological event had to be documented before the first signs of TTS, and (iii) time between first neurological symptoms and TTS diagnosis had to be < 10 days. Patients without acute neurological conditions prior to their TTS event were termed TTS and were used as controls for the Neuro-TTS group. Furthermore, patients were stratified by neurological disorders to study clinical characteristics and outcomes.

The study protocol was approved by the cantonal ethics committee Zurich (KEK-ZH Nr.: 2013-0075 and BASEC-ID 2019–02,402) and conformed to the principles of the Declaration of Helsinki. Written informed consent was waived for retrospectively included patients by the cantonal ethics committee Zurich (KEK-ZH Nr.: 2013-0075). Written informed consent was obtained from all participants included after January 1 2017 (cantonal ethics committee Zurich, BASEC ID 2019-02402).

### Data acquisition

Data were collected through review of medical charts which included information on demographics, takotsubo type (i.e. apical type and non-apical types including midventricular, basal, and focal type), clinical presentation, laboratory profile, ECG, cardiovascular risk factors, comorbidities, imaging findings from echocardiography, coronary angiography, cardiac magnetic resonance imaging, clinical management and adverse events. Furthermore, detailed data on the neurological event were collected. Symptom severity was evaluated using validated measures such as the Glasgow coma scale for intracranial hemorrhage (GCS 3–15, lower score values indicate worse status)^17^, Hunt and Hess rating scale for subarachnoid hemorrhage (SAH, 0–5, higher score values indicate greater severity)^18^, Status Epilepticus Severity Score for status epilepticus (STESS, 0–6, higher score values indicate greater severity)^19^, and National Institutes of Health Stroke Scale for ischemic stroke (NIHSS, 0–42, higher score values indicate greater severity). Information about anatomic lesion site, presumed pathophysiology of the respective diseases was obtained from medical records as well as time interval from neurological disorders to TTS diagnosis. Follow-up data collection was conducted through phone interviews, review of medical charts, or outpatients’ visits.

### Outcomes

The main outcome measure was survival at discharge. Factors associated with in-hospital mortality were assessed. Causes of in-hospital death were documented and classified as cardiovascular, non-cardiovascular, or death of unknown cause. Moreover, the incidence of major adverse cardiac and cerebrovascular events [MACCE: a composite of all-cause death, TTS recurrence, stroke or transient ischemic attack, or myocardial infarction] as well as the single components of MACCE at 10-year follow-up were studied.

### Statistical analysis

Continuous data are reported as mean ± standard deviation or medians and interquartile ranges. Categorical variables are given as numbers and percentages. Comparisons of patients’ characteristics between the Neuro-TTS group and TTS controls were performed using an unpaired t-test or the Mann–Whitney U-test for continuous data and the Pearson chi-square test or Fisher’s exact test for categorical variables. Multiple group comparisons were conducted with one-way analysis of variance or Kruskal–Wallis test. Variables that were significantly different at baseline comparison were included in a logistic regression model to identify factors associated with in-hospital mortality. Multiple imputations were conducted prior to logistic regression analysis.

Kaplan–Meier method was used to study outcome estimates, which were assessed by log-rank test. A 10-year landmark analysis was performed for patients who survived the first 30 days after the TTS event. Odds ratios (OR) are given with the corresponding 95% confidence interval (C.I.). All tests were 2-sided and statistical significance was defined as *P* < 0.05. SPSS Version 25.0 (IBM Corp., Armon, NY, USA) was used for statistical analysis and GraphPad Prism 8 (GraphPad, La Jolla, CA, USA) for figure preparation.

## Results

### Patients’ characteristics

**Figure 1:**
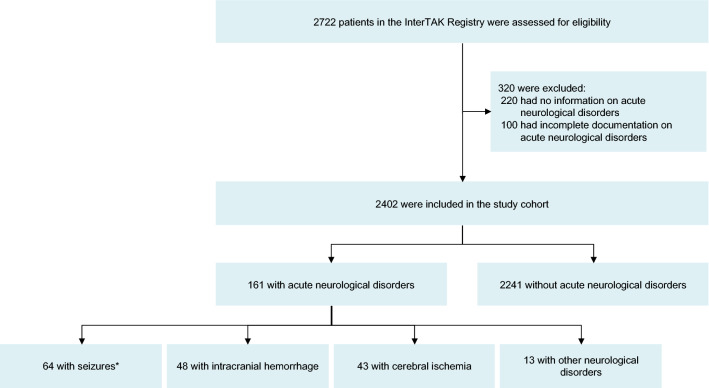
Study population. *7 patients had overlap of 2 acute neurological conditions and were classified twice: 2 with focal onset seizure and ischemic stroke, 2 with generalized onset seizure and PRES, 1 with status epilepticus and PRES, 1 with status epilepticus and SAH, and 1 with generalized onset seizure and SAH. *InterTAK Registry* International Takotsubo Registry, *PRES* Posterior reversible encephalopathy syndrome, *SAH* Subarachnoid hemorrhage.

**Figure 2:**
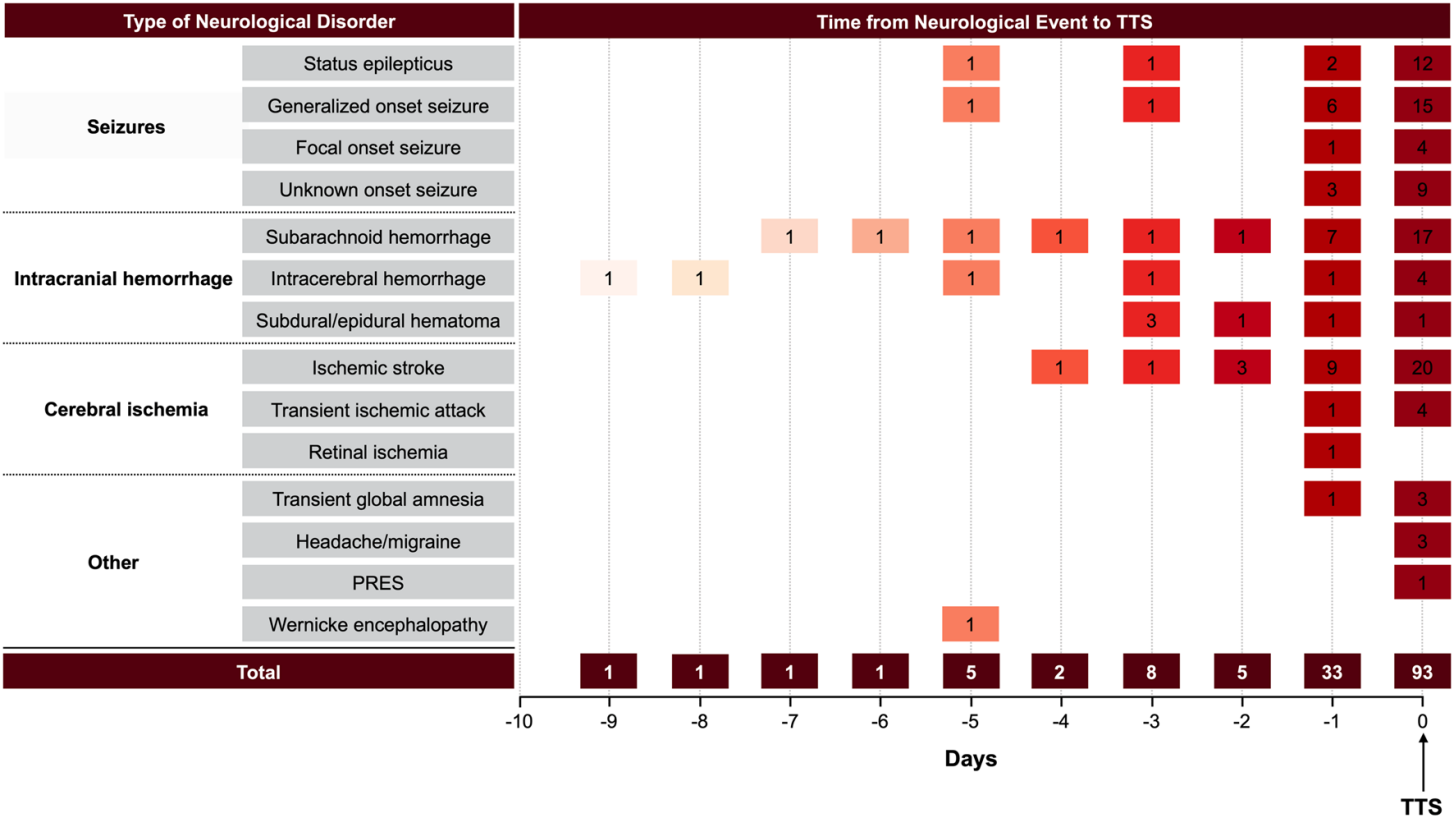
Time link between neurological disorders and takotsubo syndrome. Of the 150 patients included in the analysis median time from neurological disorders to TTS was 0 (IQR 0–1) days. Notably, time from neurological disorder to TTS was less than 2 days in 87.3% of cases. In 62% of cases the neurologic event and TTS were diagnosed on the same day, while 38% of patients were already hospitalized for the underlying neurologic conditions and TTS diagnosed during the clinical course. Numbers in boxes are the number of patients diagnosed with neurological disorders on the respective day. X-axis: days from neurological event to TTS. Y-axis: different types of neurological disorders triggering TTS. 7 patients with overlap of 2 acute neurological conditions and were excluded (2 with focal onset seizure and ischemic stroke, 2 with generalized onset seizure and PRES, 1 with status epilepticus and PRES, 1 with status epilepticus and SAH, and 1 with generalized onset seizure and SAH). In 4 cases the exact time of onset of neurological disorders was unknown (1 patient with subarachnoid hemorrhage, 1 patient with ischemic stroke, 1 patient with unknown onset seizure, and 1 patient with left frontal lobe tumor with progressive aphasia). *IQR* Interquartile range, *PRES* Posterior reversible encephalopathy syndrome, *SAH* Subarachnoid hemorrhage, *TTS* Takotsubo syndrome.

A total of 2722 patients from the InterTAK Registry with data collected between January 2011 and May 2019 were assessed for eligibility. Of these, 2402 patients had complete information on acute neurological disorders and were included in the present study. In 161 (6.7%) patients acute neurological disorders preceded the TTS event (Fig. 1). TTS was diagnosed within 2 days after neurological symptoms in 87.3% of patients (Fig. 2).

Patients’ characteristics are summarized in Table 1. Patients in the Neuro-TTS group were younger (63.7 ± 15.2 years vs. 67.5 ± 12.4 years, *P* < 0.001) and less often female (82.6% vs. 90.9%, *P* = 0.001) compared to TTS controls. Neuro-TTS patients had less often cardiac symptoms including chest pain or dyspnea. While there was no difference with regard to the frequency of the classical apical ballooning pattern (66.5% vs. 70.5%, *P* = 0.27), Neuro-TTS patients showed more often the basal TTS variant (3.7% vs. 1.0%, *P* = 0.011) on echocardiography or left ventriculography. Patients in the Neuro-TTS group had higher heart rate (91.8 ± 27.6 bpm vs. 86.9 ± 21.5 bpm, *P* = 0.022) and lower left ventricular ejection fraction (37.4 ± 11.9% vs. 41.0 ± 11.7%, *P* < 0.001) compared to TTS controls. Troponin at admission levels were comparable between Neuro-TTS and TTS controls, while troponin peak values were nearly twice as high in the Neuro-TTS group compared to TTS controls [33.91 (11.50–65.75) vs. 17.35 (6.47–41.00), factor increase of upper limit of normal, *P* < 0.001]. Furthermore, brain natriuretic peptide on admission as well as corresponding peak values were higher in the Neuro-TTS group indicating a greater degree of myocardial injury in the Neuro-TTS group.

**Table 1:** Characteristics of Takotsubo patients.

Characteristic	Neuro-TTS	TTS	*P* value
	N = 161	N = 2241	
**Demographics**			
Age—yr	63.7 ± 15.2 (N = 161)	67.5 ± 12.4 (N = 2241)	< 0.001
Female sex—no./total no. (%)	133/161 (82.6)	2036/2241 (90.9)	0.001
Body mass index—kg/m^2^	24.4 ± 5.4 (N = 102)	25.2 ± 5.3 (N = 1706)	0.17
**Takotsubo type—no./total no. (%)**			
Apical	107/161 (66.5)	1581/2241 (70.5)	0.27
Midventricular	41/161 (25.5)	538/2241 (24.0)	0.68
Basal	6/161 (3.7)	23/2241 (1.0)	0.011*
Focal	7/161 (4.3)	99/2241 (4.4)	0.97
**Symptoms on admission—no./total no. (%)**			
Chest pain	36/124 (29.0)	1487/2039 (72.9)	< 0.001
Dyspnea	34/124 (27.4)	944/2043 (46.2)	< 0.001
**Cardiac biomarkers—median (IQR)**			
Troponin on admission—factor increase in ULN°	12.42 (2.91–39.44) N = 122	10.10 (3.29–28.71) N = 1723	0.45
Troponin maximum—factor increase in ULN°	33.91 (11.50–65.75) N = 125	17.35 (6.47–41.00) N = 1799	< 0.001
Creatine kinase on admission—factor increase in ULN	1.00 (0.60–2.37) N = 99	0.88 (0.55–1.44) N = 1570	0.023
Creatine kinase maximum—factor increase in ULN	1.69 (0.71–4.67) N = 108	1.07 (0.65–1.88) N = 1648	< 0.001
BNP on admission—factor increase in ULN^†^	3.70 (1.13–10.83) N = 40	6.68 (2.11–17.76) N = 626	0.038
BNP maximum—factor increase in ULN^†^	20.61 (4.45–36.61) N = 57	9.76 (3.67–24.02) N = 822	0.032
**Inflammatory markers—median (IQR)**			
CRP on admission—mg/l	5.00 (1.95–22.50) N = 93	4.30 (1.90–14.00) N = 1468	0.39
CRP maximum—mg/l	34.00 (7.23–102.68) N = 100	10.00 (3.00–45.20) N = 1614	< 0.001
WBC on admission	11.40 (8.80–16.62) N = 139	9.70 (7.50–12.59) N = 1907	< 0.001
WBC maximum	12.70 (10.00–18.32) N = 145	10.51 (8.26–13.53) N = 1967	< 0.001
**First ECG—no./total no. (%)**			
Sinus rhythm	114/123 (92.7)	1853/1990 (93.1)	0.85
Atrial fibrillation	6/123 (4.9)	121/1990 (6.1)	0.59
ST-segment elevation	53/124 (42.7)	757/1984 (38.2)	0.31
ST-segment depression	12/122 (9.8)	152/1980 (7.7)	0.39
T-wave inversion	41/122 (33.6)	854/1982 (43.1)	0.040
Left bundle branch block	2/122 (1.6)	102/1980 (5.2)	0.08
QTc—ms	468.1 ± 45.6 (N = 103)	458.8 ± 47.0 (N = 1513)	0.052
**Hemodynamics—mean ± SD (N)**			
Heart rate—beats/min	91.8 ± 27.6 (N = 114)	86.9 ± 21.5 (N = 1737)	0.022
Systolic blood pressure—mm Hg	127.8 ± 31.4 (N = 119)	130.7 ± 29.4 (N = 1720)	0.31
Diastolic blood pressure—mm Hg	76.6 ± 19.6 (N = 118)	76.5 ± 16.6 (N = 1684)	0.97
Left ventricular ejection fraction—%^‡^	37.4 ± 11.9 (N = 141)	41.0 ± 11.7 (N = 1942)	< 0.001
Left ventricular end-diastolic pressure—mm Hg	20.5 ± 8.7 (N = 69)	21.9 ± 8.0 (N = 1153)	0.17
**Cardiovascular risk factors—no./total no. (%)**			
Hypertension	87/153 (56.9)	1453/2199 (66.1)	0.02
Diabetes mellitus	9/154 (5.8)	352/2212 (15.9)	0.001
Current smoking	37/145 (25.5)	405/2119 (19.1)	0.029
Hypercholesterolemia	35/151 (23.2)	720/2167 (33.2)	0.011
Positive family history	19/127 (15.0)	370/1898 (19.5)	0.21
**Comorbidities—no./total no. (%)**			
COPD or Asthma	20/149 (13.4)	361/2118 (17.0)	0.25
Cancer (total)	33/147 (22.4)	363/2093 (17.3)	0.12
Hyperthyroidism	5/150 (3.3)	91/2181 (4.2)	0.62
Hypothyroidism	22/150 (14.7)	338/2182 (15.5)	0.79
**Medication on admission—no./total no. (%)**			
** Cardiovascular medication**			
ACE inhibitor or ARB	38/110 (34.5)	673/1748 (38.5)	0.41
Beta-blocker	30/110 (27.3)	481/1748 (27.5)	0.96
Calcium-channel antagonist	11/110 (10.0)	164/1737 (9.4)	0.85
Statin	20/110 (18.2)	411/1737 (23.7)	0.19
Aspirin	31/110 (28.2)	508/1737 (29.2)	0.81
P2Y_12_ antagonist	8/110 (7.3)	108/1737 (6.2)	0.66
Coumarin	4/110 (3.6)	76/1737 (4.4)	1.0*
**Acute cardiac care treatment—no./total no. (%)**			
Invasive or noninvasive ventilation	75/158 (47.5)	340/2220 (15.3)	< 0.001
Cardiopulmonary resuscitation	25/157 (15.9)	159/2232 (7.1)	< 0.001
Catecholamine use	48/158 (30.4)	260/2219 (11.7)	< 0.001
**In-hospital complications—no./total no. (%)**			
Cardiogenic shock	24/159 (15.1)	191/2211 (8.6)	0.006
Death	28/161 (17.4)	90/2241 (4.0)	< 0.001
**10-year outcome—no./total no. (%)**			
MACCE	52/161 (32.3)	347/2241 (15.5)	< 0.001
Death	40/161 (24.8)	224/2241 (10.0)	< 0.001
Recurrence	4/161 (2.5)	69/2241 (3.1)	0.66
Stroke/TIA	10/161 (6.2)	58/2241 (2.6)	< 0.001
Myocardial infarction	0/161 (0.0)	18/2241 (0.8)	0.35

*ACE* Angiotensin-converting-enzyme, *ARB* Angiotensin-receptor blocker, *BNP* Brain natriuretic peptide, *COPD* Chronic obstructive pulmonary disease, *CRP* c-reactive protein, *ECG* Electrocardiogram, *IQR* Interquartile range, *MACCE* major adverse cardiac and cerebrovascular event, *QTc* QT interval corrected for heart rate, *SD* Standard deviation, *TIA* transient ischemic attack, *TTS* Takotsubo syndrome, *ULN* Upper limit of the normal, *WBC* white blood cell count.

°Including upper limits of the normal range for troponin T, high-sensitivity troponin T, and troponin I.

^†^Including upper limits of the normal range for brain natriuretic peptide and the N-terminal of prohormone brain natriuretic peptide.

^‡^Data obtained during catheterization or echocardiography if both results were available data from catheterization were used.

*Fisher’s exact test.

### Clinical spectrum of neurological disorders

Of the 161 patients with neurological disorders, the most common neurological disorders were seizures (N = 64, 39.8%; Fig. 1, Table 2), intracranial hemorrhage (N = 48, 29.8%, Fig. 1, Table 3) and cerebral ischemia (N = 43, 26.7%, Fig. 1, Table 4). Transient global amnesia was identified in 4 patients, posterior reversible encephalopathy syndrome (PRES) in 4 patients, migraine or headache disorders in 3 patients, intracranial tumor with progressive aphasia upon presentation in 1 patient, and Wernicke encephalopathy in 1 patient (Supplementary Table 1). The proportion of males was significantly higher in patients with intracranial hemorrhage than in patients with seizures or cerebral ischemia, and patients with intracranial hemorrhage and seizures were younger than patients with cerebral ischemia (Table 5 and Supplementary Fig. 1). Left ventricular ejection fraction was more reduced in patients with intracranial hemorrhage, while left ventricular end-diastolic pressure was substantially higher compared to patients with seizures and cerebral ischemia. Characteristics of patients stratified by neurologic disorders are summarized in Table 5. The prevalence of seizure/epilepsy and subarachnoid hemorrhage seems to be higher in the InterTAK Registry than in the general population using data Global Burden of Disease statistics^20^, while numbers of ischemic stroke and intracerebral hemorrhage were similar in both cohorts (Supplementary Fig. 2).

**Table 2:** Classification of seizures according to type, semiology, and pathophysiology.

Seizures N = 64			
*Type*			
Status epilepticus N = 18	Generalized onset seizure N = 26	Focal onset seizure N = 7	Unknown onset seizure N = 13
*Semiology*			
N = 11 Convulsive N = 3 Non-Convulsive N = 2 Focal N = 1 Aware N = 1 Non-aware N = 2 Unknown STESS > 2 points: 8/18 (44.4%)	N = 26 Motor Tonic–clonic	N = 5 Focal to bilateral tonic–clonic N = 1 Impaired awareness N = 1 Aware, motor onset	N = 12 Motor Tonic–clonic or other motor N = 1 Unclassified
*Pathophysiology*			
N = 8 History of seizure N = 7 Acute symptomatic N = 12 Structural lesions N = 6 Right hemispheric involvement	N = 6 History of seizure N = 11 Acute symptomatic N = 13 Structural lesions^#^ N = 8 Right hemispheric involvement	N = 4 History of seizure N = 2 Acute symptomatic N = 4 Structural lesions N = 3 Right hemispheric involvement	N = 4 History of seizure N = 8 Structural lesions N = 5 Right hemispheric involvement

*STESS* Status epilepticus severity score.

^#^Data on structural lesions were available in 24 patients.

**Table 3:** Classification of intracranial hemorrhage according to type, clinical severity, and pathophysiology.

Intracranial hemorrhage N = 48		
*Type*		
Subarachnoid hemorrhage N = 33	Intracerebral hemorrhage N = 9	Subdural/epidural hematoma N = 6
*Clinical severity*		
Glasgow Coma Scale median 7 points (IQR 3–12 points, N = 28)	Glasgow Coma Scale median 9 points (IQR 5–14 points, N = 8)	Glasgow Coma Scale median 14 points (IQR 9–15 points, N = 6)
Hunt and Hess Scale median grade 4 (IQR, 2–5, N = 24)		
*Pathophysiology*		
N = 27 Aneurysmal Site of Aneurysm: AcomA or ACA (N = 12) Right MCA (N = 4) Right PcomA (N = 2) Basilar artery (N = 2) Right ICA (N = 1) Left MCA (N = 1) PICA (N = 1) Multiple sites incl. AcomA (N = 2) Unknown (N = 2) N = 5 Traumatic N = 1 Unknown	N = 8 Spontaneous N = 1 Traumatic Localization: IVH (N = 6)* Lobar (N = 4)* Deep (N = 2)* Lobar/deep with right hemispheric involvement (4/6) Infratentorial (N = 2)*	N = 4 Traumatic Localization: Supratentorial (N = 6) Right hemispheric involvement (4/6)

*ACA* Anterior cerebral artery, *AcomA* Anterior communicating artery, *ICA* Internal carotid artery, *IQR* Interquartile range, *IVH* Intraventricular hemorrhage, *MCA* Middle cerebral artery, *PcomA* Posterior communicating artery, *PICA* Posterior inferior cerebellar artery.

*Data on localization were available in 8 patients.

**Table 4:** Classification of cerebral ischemia according to type, clinical severity, and vascular territory.

Cerebral ischemia N = 43		
*Type*		
Ischemic stroke N = 37	Transient ischemic attack N = 5	Retinal ischemia N = 1
*Clinical severity*		
NIHSS on admission median 6 points (IQR 2–14 points, N = 35)	NIHSS on admission median 0 points (IQR 0–2 points, N = 5)	Amaurosis
*Vascular territory*		
N = 11 Right anterior circulation N = 10 Left anterior circulation N = 8 Posterior circulation N = 7 Multiple territories N = 1 Unknown, but definite anterior circulation	N = 3 Left anterior circulation N = 1 Right anterior circulation N = 1 Unknown	Left-sided amaurosis

*NIHSS* National Institutes of Health stroke scale.

**Table 5:** Characteristics of Takotsubo patients stratified by neurological disorders.

Characteristic	Seizures	Cerebral ischemia	Intracranial hemorrhage	*P* value
	N = 57	N = 41	N = 46	
**Demographics**				
Age—yr	61.8 ± 13.2 (N = 57)	72.2 ± 13.6 (N = 41)	58.6 ± 17.0 (N = 46)	< 0.001
Female sex—no./total no. (%)	52/57 (91.2)	32/41 (78.0)	33/46 (71.7)	0.035
Body mass index—kg/m^2^	24.1 ± 5.6 (N = 39)	23.9 ± 4.2 (N = 24)	24.3 ± 5.1 (N = 25)	0.95
**Takotsubo type—no./total no. (%)**				
Apical	37/57 (64.9)	30/41 (73.2)	32/46 (69.6)	0.68
Midventricular	15/57 (26.3)	8/41 (19.5)	10/46 (21.7)	0.71
Basal	2/57 (3.5)	1/41 (2.4)	3/46 (6.5)	0.60
Focal	3/57 (5.3)	2/41 (4.9)	1/46 (2.2)	0.71
**Symptoms on admission—no./total no. (%)**				
Chest pain	10/44 (22.7)	10/38 (26.3)	8/28 (28.6)	0.85
Dyspnea	16/45 (35.6)	8/37 (21.6)	5/28 (17.9)	0.18
**Cardiac biomarkers—median (IQR)**				
Troponin on admission—factor increase in ULN°	5.97 (2.45–18.73) N = 44	25.43 (5.00–55.00) N = 29	16.73 (3.11–60.76) N = 38	0.06
Troponin maximum—factor increase in ULN°	25.00 (7.42–44.04) N = 45	40.01 (20.29–70.22) N = 31	37.25 (12.90–86.00) N = 38	0.10
Creatine kinase on admission—factor increase in ULN	1.24 (0.52–3.24) N = 37	1.26 (0.68–2.66) N = 27	0.99 (0.70–2.70) N = 25	0.78
Creatine kinase maximum—factor increase in ULN	1.99 (0.77–5.78) N = 41	1.81 (0.92–4.40) N = 29	1.35 (0.70–4.59) N = 27	0.71
BNP on admission—factor increase in ULN^†^	3.56 (0.78–8.32) N = 13	6.17 (2.07–48.07) N = 12	2.15 (0.64–9.51) N = 12	0.09
BNP maximum—factor increase in ULN^†^	21.18 (4.17–37.22) N = 21	19.38 (6.04–40.44) N = 15	20.71 (2.87–59.21) N = 17	0.92
**Inflammatory markers—median (IQR)**				
CRP on admission—mg/l	6.40 (1.65–29.50) N = 37	7.35 (4.45–19.60) N = 28	2.05 (1.25–14.00) N = 20	0.08
CRP maximum—mg/l	35.05 (8.34–80.53) N = 40	36.85 (9.98–112.7) N = 28	72.00 (12.30–111.8) N = 21	0.68
WBC on admission	11.90 (9.40–15.90) N = 40	10.50 (8.04–13.63) N = 36	15.00 (10.00–19.40) N = 39	0.014
WBC maximum	13.20 (9.99–17.15) N = 54	11.90 (9.87–14.24) N = 37	18.12 (11.30–21.70) N = 39	0.007
**First ECG—no./total no. (%)**				
Sinus rhythm	40/41 (97.6)	29/34 (85.3)	32/34 (94.1)	0.12
Atrial fibrillation	1/41 (2.4)	3/34 (8.8)	1/34 (2.9)	0.36
ST-segment elevation	15/42 (35.7)	19/34 (55.9)	16/34 (47.1)	0.21
ST-segment depression	4/41 (9.8)	3/34 (8.8)	4/33 (12.1)	0.90
T-wave inversion	15/41 (36.6)	13/34 (38.2)	9/33 (27.3)	0.59
Left bundle branch block	0/41 (0.0)	0/34 (0.0)	1/33 (3.0)	0.32
QTc—ms	467.1 ± 44.7 (N = 36)	457.7 ± 37.5 (N = 24)	474.0 ± 41.7 (N = 29)	0.37
**Hemodynamics—mean ± SD (N)**				
Heart rate—beats/min	98.9 ± 28.6 (N = 41)	83.4 ± 21.2 (N = 26)	93.8 ± 29.2 (N = 33)	0.08
Systolic blood pressure—mm Hg	123.1 ± 27.6 (N = 42)	135.0 ± 34.7 (N = 30)	126.0 ± 31.5 (N = 33)	0.27
Diastolic blood pressure—mm Hg	74.9 ± 22.0 (N = 42)	74.9 ± 17.5 (N = 30)	78.0 ± 15.6 (N = 32)	0.74
Left ventricular ejection fraction—%^‡^	41.0 ± 10.7 (N = 53)	35.3 ± 10.7 (N = 37)	34.1 ± 12.1 (N = 36)	0.009
Left ventricular end-diastolic pressure—mm Hg	18.8 ± 7.6 (N = 31)	19.6 ± 8.4 (N = 16)	26.0 ± 9.5 (N = 12)	0.039
**Cardiovascular risk factors—no./total no. (%)**				
Hypertension	28/57 (49.1)	25/40 (62.5)	22/40 (55.0)	0.43
Diabetes mellitus	6/57 (10.5)	2/40 (5.0)	0/40 (0.0)	0.09
Current smoking	13/55 (23.6)	5/35 (14.3)	13/39 (33.3)	0.16
Hypercholesterolemia	16/57 (28.1)	10/39 (25.6)	6/39 (15.4)	0.34
Positive family history	8/49 (16.3)	5/32 (15.6)	4/34 (11.8)	0.84
**Comorbidities—no./total no. (%)**				
COPD or Asthma	9/56 (16.1)	5/39 (12.8)	2/38 (5.3)	0.28
Cancer (total)	18/56 (32.1)	6/39 (15.4)	5/37 (13.5)	0.052
Hyperthyroidism	4/56 (7.1)	0/40 (0.0)	0/38 (0.0)	0.06
Hypothyroidism	9/56 (16.1)	7/40 (17.5)	5/38 (13.2)	0.87
**Medication on admission—no./total no. (%)**				
**Cardiovascular medication**				
ACE inhibitor or ARB	12/41 (29.3)	14/28 (50.0)	6/29 (20.7)	0.051
Beta-blocker	12/41 (29.3)	9/28 (32.1)	7/29 (24.1)	0.79
Calcium-channel antagonist	5/41 (12.2)	4/28 (14.3)	2/29 (6.9)	0.66
Statin	9/41 (22.0)	7/28 (25.0)	3/29 (10.3)	0.32
Aspirin	14/41 (34.1)	11/28 (39.3)	4/29 (13.8)	0.08
P2Y_12_ antagonist	6/41 (14.6)	2/28 (7.1)	0/29 (0.0)	0.09
Coumarin	1/41 (2.4)	1/28 (3.6)	2/29 (6.9)	0.64
**Acute cardiac care treatment—no./total no. (%)**				
Invasive or noninvasive ventilation	25/55 (45.5)	10/40 (25.0)	35/46 (76.1)	< 0.001
Cardiopulmonary resuscitation	10/55 (18.2)	3/40 (7.5)	10/46 (21.7)	0.18
Catecholamine use	12/55 (21.8)	8/40 (20.0)	25/46 (54.3)	< 0.001
**In-hospital complications—no./total no. (%)**				
Cardiogenic shock	10/56 (17.9)	4/40 (10.0)	7/46 (15.2)	0.56
Death	5/57 (8.8)	6/41 (14.6)	14/46 (30.4)	0.013

Analysis excludes patients who had overlap of 2 neurological disorders: focal onset seizure and ischemic stroke (N = 2), generalized onset seizure and posterior reversible encephalopathy syndrome (N = 2), status epilepticus and posterior reversible encephalopathy syndrome (N = 1), status epilepticus and subarachnoid hemorrhage (N = 1) and generalized onset seizure and subarachnoid hemorrhage (N = 1).

*ACE* Angiotensin-converting-enzyme, *ARB* Angiotensin-receptor blocker, *BNP* Brain natriuretic peptide, *COPD* Chronic obstructive pulmonary disease, *CRP* c-reactive protein, *ECG* Electrocardiogram, *IQR* Interquartile range, *QTc* QT interval corrected for heart rate, *SD* Standard deviation, *ULN* Upper limit of the normal, *WBC* white blood cell count.

°Including upper limits of the normal range for troponin T, high-sensitivity troponin T, and troponin I.

^†^Including upper limits of the normal range for brain natriuretic peptide and the N-terminal of prohormone brain natriuretic peptide.

^‡^Data obtained during catheterization or echocardiography if both results were available data from catheterization were used.

### Characteristics of patients with seizures

Of the 64 patients who had TTS secondary to seizures, 18 had status epilepticus (STESS > 2 points in 44.4%), 26 had generalized onset seizures, 7 had focal onset seizures, and 13 had unknown onset seizures (Table 2). A history of seizure was identified in 22 patients and 17 patients were on antiepileptic drug treatment prior to TTS event. Structural underlying brain lesions were present in 37 patients (data available in N = 62) and involved the right hemisphere in 22 (59.5%) patients. Seizures were considered acute symptomatic in 20 patients, including patients with SAH (N = 2), ischemic stroke (N = 3), and PRES (N = 3). Younger age and lower predominance of females seemed to be more pronounced after status epilepticus and generalized onset seizures than after seizures with focal onset or unknown onset seizure (Supplementary Fig. 1). An atypical TTS variant was observed in 37.5% after status epilepticus and 43.5% after generalized onset seizures.

### Characteristics of patients with intracranial hemorrhage

Of the 48 patients who had TTS secondary to intracranial hemorrhage, 33 had SAH, 9 intracerebral hemorrhage, and 6 subdural or epidural hematoma. Mean age of patients with SAH was 58.8 ± 18.0 years, 77.4% were female (Supplementary Fig. 1), and 35.5% had an atypical TTS variant. Clinical severity of SAH was severe (median GCS 7 points and median Hunt and Hess grade 4) and 81.8% (27 / 33) of SAH were caused by aneurysmatic origin. Site of aneurysm included the anterior communicating artery or anterior cerebral artery in 12 out of 27 (44.4%) cases. Intracerebral hemorrhage occurred spontaneously in 8 out of 9 cases with overall moderate clinical severity (median GCS 9 points). Subdural or epidural hematoma were located supratentorial in all cases and had traumatic origin in 66.7% (4 / 6) of cases (Table 3).

### Characteristics of patients with cerebral ischemia

Of the 43 patients with cerebral ischemia, 37 had ischemic stroke, 5 transient ischemic attack and 1 retinal ischemia. Mean age of patients with ischemic stroke was 71.6 ± 14.2 years, 77.1% were female, and 25.7% had an atypical TTS variant. Clinical stroke severity of patients with stroke was moderate (median NIHSS 6 points). Anterior circulation stroke was documented in 21 (56.8%) patients of which 11 were right-sided and 10 were left-sided. Involvement of the insular cortex was documented in 8 out of 22 patients (36.4%), and evidence of an embolic stroke origin was observed in 29 out of 32 patients (90.6%) with available descriptive imaging data, respectively. Transient ischemic attack affected the anterior circulation in 4 out of 5 of cases (Table 4).

### Clinical course and outcomes

Patients in the Neuro-TTS group more often required non-invasive or invasive ventilation (47.5% vs. 15.3%, *P* < 0.001), catecholamine administration (30.4% vs. 11.7%, *P* < 0.001) and cardiopulmonary resuscitation (15.9% vs. 7.1%, *P* < 0.001) compared to TTS controls.

**Figure 3:**
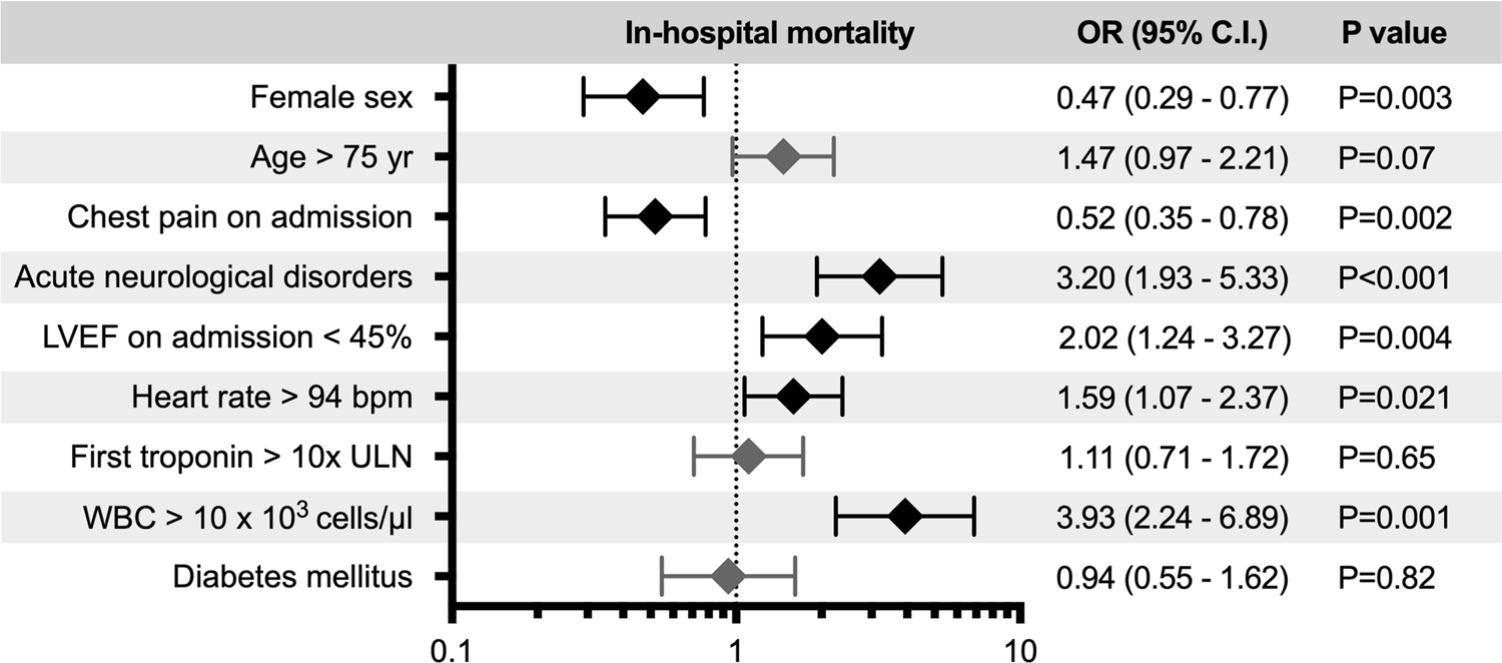
Factors associated with in-hospital mortality. Acute neurological disorders, left ventricular ejection fraction < 45%, heart rate over 94 bpm, and WBC > 10 × 10^3^ cells/µ were identified as factors associated with in-hospital mortality. Bpm beats per minute; C.I. confidence interval; LVEF left ventricular ejection fraction; OR odds ratio; ULN upper limit of normal; WBC white blood cell count. Errors bars indicate 95% confidence intervals. Black indicates statistically significant variables, while grey is not significant.

Proportions of cardiogenic shock (15.1% vs. 8.6%, *P* = 0.006) and in-hospital mortality (17.4% vs. 4.0%, *P* < 0.001) were substantially higher in Neuro-TTS patients (Table 1). When comparing causes of death, cardiovascular causes of death were substantially higher in Neuro-TTS group than in TTS controls (82.1% vs. 47.8%, *P* = 0.005, Supplementary Fig. 3). On multivariable logistic regression the presence of acute neurological disorders (OR 3.20, 95% C.I. 1.93–5.33, *P* < 0.001) was associated with increased in-hospital mortality (Fig. 3). 10-year outcome analysis demonstrated increased MACCE (32.3% vs. 15.5%, *P* < 0.001) and mortality (24.8% vs. 10.0%, *P* < 0.001) rates in TTS patients with neurological disorders, while recurrence rates were not statistically significant different in the neuro-TTS group and in TTS controls (2.5% vs. 3.1%, *P* = 0.66, Table 1). Moreover, we conducted a 10-year landmark analysis with a landmark set at 30-days. Landmark analysis showed substantially higher mortality within the 30 days after admission in patients with neurological disorders. After the landmark including only patients alive 30 days after TTS, patients with neurological disorders showed substantially higher long-term mortality compared to TTS controls without neurological disorders (*P* = 0.020, Supplementary Fig. 4).

## Discussion

The present study based on data from the InterTAK Registry demonstrates that neurological disorders are common triggering factors of TTS. TTS patients with neurological disorders exhibit a distinct clinical profile including younger age, lower female predominance, and less clinically apparent cardiac symptoms at presentation. Furthermore, patients with neurological disorders have more pronounced myocardial injury as reflected by markedly elevated cardiac biomarkers and more impaired left ventricular ejection fraction compared to patients without neurological disease. Seizures, intracerebral hemorrhage, and ischemic stroke constitute the most common neurological disorders preceding TTS.

The presence of cardiac disorders is frequently observed after neurological disorders and is linked to a high burden of morbidity and mortality^1–4^. Cardiac complications after acute neurological disorders comprise a wide spectrum ranging from benign ECG alterations, cardiac biomarker abnormalities, myocardial disorders, left ventricular dysfunction, malignant arrhythmias to sudden cardiac death^1,2,6,21^. The link between TTS and neurovascular events was underscored more than two decades ago. For instance, the first version of the Mayo Clinic Diagnostic Criteria excluded the presence of neurologic disorders for diagnosis of TTS^22^. Instead the term neurogenic stunned myocardium was frequently found in the literature to describe myocardial dysfunction after acute neurological injury especially after subarachnoid hemorrhage^23,24^. Over the last years, the interplay of the brain and heart has gained growing interest in the medical community and TTS is now acknowledged as a cardiac consequence of acute neurological events^2,6,25–27^. While the understanding of the precise pathomechanisms underlying TTS development remain incomplete, there is evidence that autonomic imbalance with excessive catecholamine release and increased cardiomyocyte response to catecholamines infer the pathophysiology^28–30^. Levels of catecholamines are reported to be 4 times higher in TTS than in patients with myocardial infarction even three to five days after hospital admission, and over 30 times higher than normal resting values^29^. The plasma half-lives of epinephrine and norepinephrine are approximately 1–3 min^31^. Thus, the maximum catecholamine levels at symptom onset will be substantially higher than any measurement taken on hospital admission considering an onset-to-door time of at least 30 min^32^. Prolonged elevation of circulating catecholamines for several hours has also been reported after acute neurological disorders such as seizures and stroke^33,34^.

Cardiovascular functions are controlled by a complex network of cortical and subcortical forebrain regions (including the amygdala, the insula, the hippocampus, as well as lateral and mesial frontal regions)^1,2,35^. Neuro-imaging studies on TTS patients have demonstrated altered function of brain regions which are responsible for autonomic regulation^10^.

In our cohort, one out of 15 TTS patients had a preceding acute neurological disorder as the underlying triggering factor. The prevalence of seizure/epilepsy and subarachnoid hemorrhage in our study population seems to be higher than in the general population using data from Global Burden of Disease statistics, while numbers of ischemic stroke and intracerebral hemorrhage were similar in both cohorts^20^. Elderly women after an emotional triggering factor (e.g. death of a beloved one) have historically been considered as the high-risk population for TTS. This is in contrast to patients with TTS after neurological disorders who are younger and more frequently male than expected. It has been suggested that TTS development depends on the degree of sympathetic stimulation^36^. This could explain the atypical clinical profile of TTS patients with neurological disorders. It may be speculated that younger individuals and males may require a more intense trigger with greater sympathetic stimulation, while only a mild trigger with low sympathetic stimulation might be sufficient to provoke TTS in elderly. The way acute neurological disorders affect the function of the central autonomic network differs among neurological disorders. In seizures and status epilepticus there is paroxysmal stimulation of the autonomic nervous system and in SAH there is an early intense sympathetic stimulation, which directly affects the hypothalamus pituitary-adrenal axis via the amygdala-insular complex. The presence of ischemic stroke lesions, on the other hand, mainly leads to loss of function or network dysfunction.

TTS secondary to seizures occurred most often after generalized onset (i.e., bilateral tonic–clonic), unknown onset motor seizures or focal to bilateral tonic–clonic seizures. In these seizure types, it has been shown that the postictal phase is dominated by sympathetic overactivation and increased heart rate with parasympathetic suppression^37,38^. In contrast, focal unaware seizures (complex partial seizures), which were rather uncommon in our cohort, were associated with ictal asystole or bradycardia^39^. Moreover, we observed that nearly 60% of structural brain lesions in patients with TTS secondary to seizures involved the right hemisphere, which may be of interest as right hemispheric lesions may promote susceptibility to autonomic dysfunction^40–42^.

Observational studies have demonstrated that TTS secondary to SAH occurs in approximately 10–15% of patients and that severity of SAH according to Hunt and Hess Scale can predict the occurrence of cardiac dysfunction^24,43^. This corroborates the findings of the present study as virtually all patients had severe SAH as reflected by low GCS and high Hunt and Hess Scale. Interestingly, more than half of registered SAH cases were related to ruptured anterior communicating artery aneurysms.

In our study, 36% of patients with ischemic stroke had involvement of the insular cortex. Insular cortex damage has been associated with increased sympathetic nervous system activity and cardiovascular system dysregulations including arrhythmias or myocardial injury^42,44,45^. Moreover, previous studies investigating TTS secondary to ischemic stroke have suggested that hemispheric lesions and especially insular cortex involvement may promote TTS occurrence^46,47^. Median NIHSS in these series was 16, which was higher than observed herein. Thus, our results indicate that TTS secondary to ischemic stroke may also be observed in patients with overall lower stroke severity. In our cohort, TTS has been diagnosed early after SAH, while after subdural/epidural hematoma TTS has been seen after a median of 3 days. Clinical severity was substantially higher in patients with SAH or intracerebral hemorrhage than in patients with subdural or epidural hematoma. Therefore, it can be likely that clinical disease severity of the underlying neurological disorder might impact time onset of TTS.

Patients with neurological disorders had a higher prevalence of the basal TTS phenotype. The basal TTS form constitutes a relatively rare morphological TTS variant and has been linked to neurological disorders such as SAH^8,48^. Furthermore, it has been suggested that the left ventricular apex is spared out after neurological disorders^49^. However, our findings challenge the concept of left ventricular apical-sparing as we have identified the apical TTS form in more than two-thirds of TTS patients with primary neurological disorders.

TTS secondary to neurological disorders was associated with increased rates of adverse events and in-hospital mortality. After controlling for major confounders, the presence of acute neurological disorders was associated with increased in-hospital mortality. This again highlights the importance to consider TTS patients with primary acute neurological conditions as a high-risk population despite younger age and less pre-existing cardiovascular risk factors.

Although TTS has gained heightened awareness during the last years, it likely remains an underdiagnosed and underreported disorder. Especially in patients with acute neurological diseases, TTS may remain clinically silent. Classical clinical symptoms of TTS such as chest pain or dyspnea were less often documented in the Neuro-TTS group compared with TTS controls. In addition, patients with neurological disorders had an unfavorable clinical course and therefore it might be even considered to actively screen for elevation of cardiac biomarkers and signs of heart failure. Therefore, a rise in sensitive biomarkers of myocardial disorders or dysfunction (i.e. cardiac troponin or BNP, respectively) should prompt cardiac evaluation. Additionally, prolonged clinical monitoring and non-invasive cardiac investigations should be considered and treatment with catecholamines or QT-prolonging drugs should be avoided if possible given the putative involvement in the pathogenesis.

We have shown that acute neurological disorders ranging from benign to life-threatening can provoke TTS. TTS patients with neurological disorders have substantial rates of adverse events. Outcome analysis demonstrated increased mortality in the first 30 days after the TTS event in patients with neurological disorders. The increased mortality rates within the first 30 days are likely driven by the underlying neurological state and TTS adding a secondary hit. Our findings emphasize the need of a standardized cardiac work-up in patients with acute neurological disorders to avoid underdiagnosing of TTS. The considerable rates of adverse events in patients with neurological disorders and TTS should prompt neurologist to heightened awareness of the syndrome.

## Limitations

This study has inherent limitations of observational studies which do not allow to establish causal relationships. However, this study design can be particularly valuable in understudied and low-incidence diseases such as TTS. A core-team of physicians and clinical scientists reviewed all medical records and imaging data centrally to ensure correct diagnosis and data quality. However, detailed neuroimaging data beyond description of pathological findings were not available in a substantial proportion of cases. The true prevalence of TTS in patients with neurological disorders is likely to be higher as TTS might have remained clinically silent or undetected especially in elderly or in patients with milder neurological disorders.

## Supplementary Information

Supplementary Information.
