# Supplementary material for: Clinical correlates and prognostic impact of neurologic disorders in Takotsubo syndrome
Source: Sci Rep. 2021 Dec 7;11:23555. doi: 10.1038/s41598-021-01496-9 (PMC8651780; doi:10.1038/s41598-021-01496-9)
Supplement: Supplementary file 1 — Supplementary Information. [file 41598_2021_1496_MOESM1_ESM.pdf]

Supplementary Appendix

Supplementary Table 1. Additional neurological triggers for takotsubo syndrome.

	Age (yrs.)	Female sex	Clinical presentation
**Transient global amnesia** N=4	73.3 ± 7.0	100% (N=4)	N= 4 Amnesia
**PRES** N=4	49.3 ± 12.9	50.0% (N=2)	N= 2 Generalized onset seizure<br>N= 1 Status epilepticus<br>N= 1 Unclear consciousness after fall
**Headache / Migraine** N=3	67.7 ± 0.9	100% (N=3)	N= 1 Migraine without aura<br>N= 1 Headache with features of migraine<br>N= 1 Headache due to zoster neuralgia
**Intracranial tumor** N=1	67	100% (N=1)	Left frontal lobe tumor with progressive aphasia
**Wernicke encephalopathy** N=1	58	100% (N=1)	Hypovitaminosis and hyperactive delirium

Transient global amnesia, PRES, headache or migraine, intracranial tumors and Wernicke encephalopathy represent additional neurological disorders which can precipitate takotsubo syndrome.
PRES posterior reversible encephalopathy syndrome.

Supplementary Figure 1. Age and sex distribution.

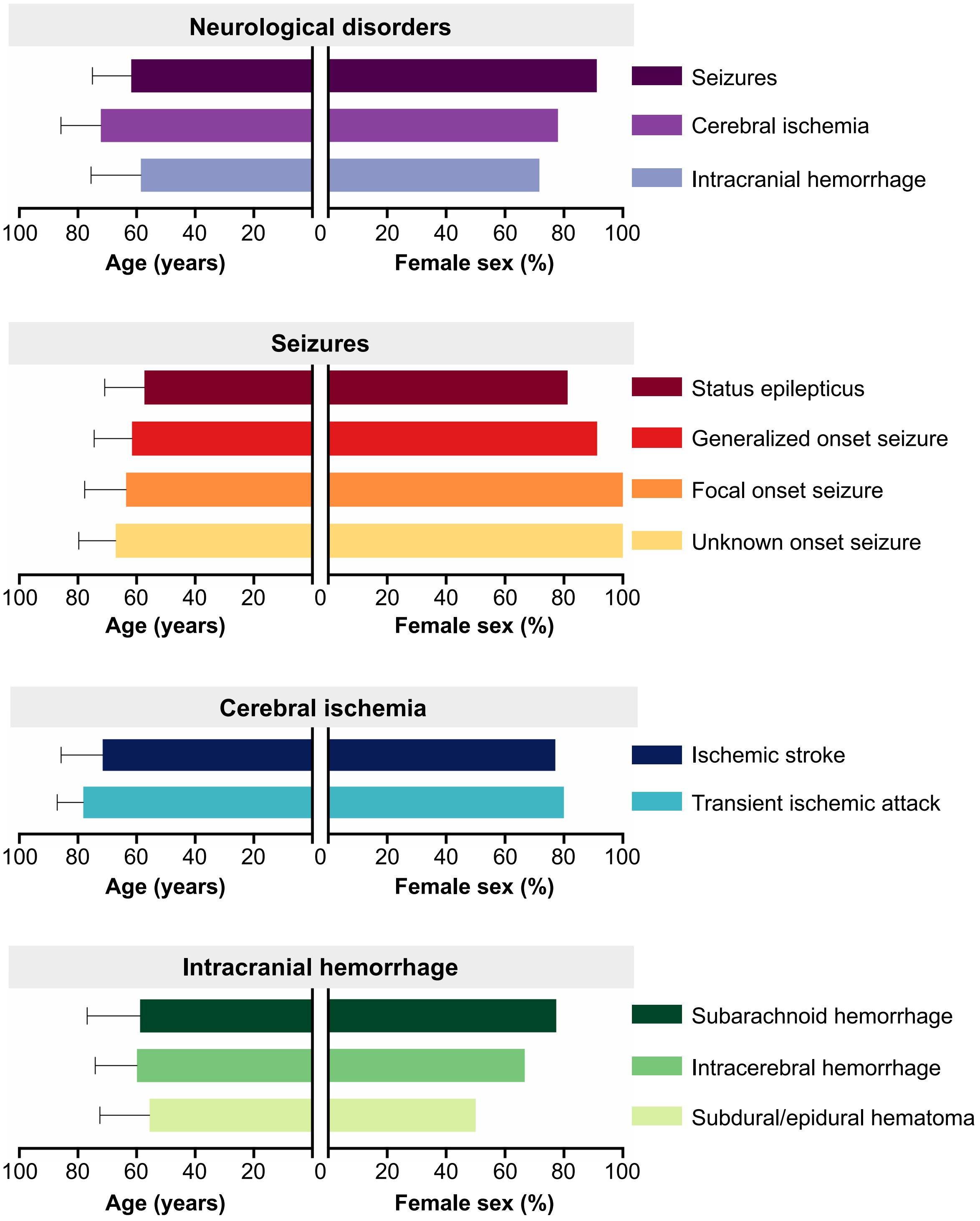

Age (left) and sex (right) distribution among takotsubo syndrome patients with neurological disorders.

Analysis excludes patients who had overlap of 2 neurological disorders: 2 with focal onset seizure and ischemic stroke, 2 with generalized onset seizure and PRES, 1 with status epilepticus and PRES, 1 with status epilepticus and SAH, and 1 with generalized onset seizure and SAH.

PRES posterior reversible encephalopathy syndrome; SAH subarachnoid hemorrhage.

Supplementary Figure 2. Prevalence of neurological disorders.

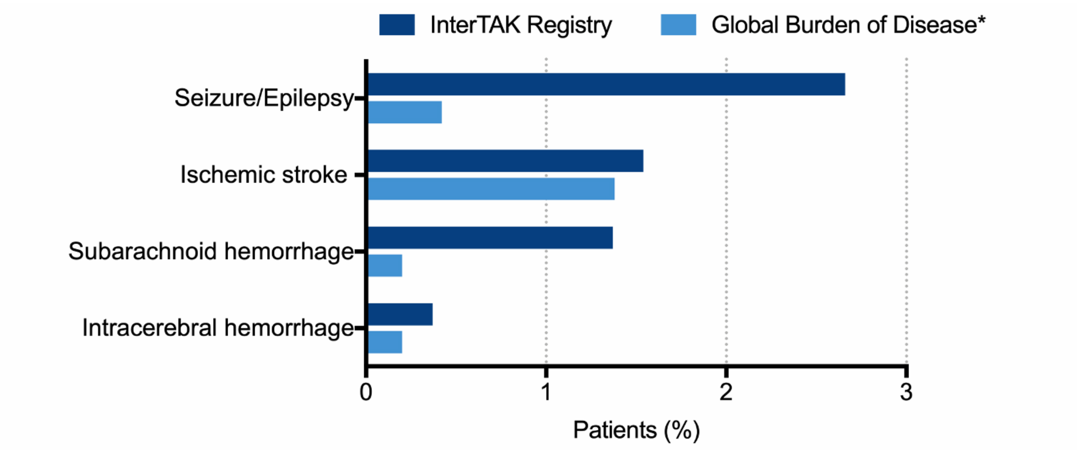

	InterTAK Registry		Global Burden of Disease*	
Population	N=2402		≈ 526 mio.	
	**No. of patients**	**Prevalence**	**No. of patients**	**Prevalence**
Seizure/Epilepsy	64	2.66%	2217896	0.42%
Ischemic stroke	37	1.54%	7271210	1.38%
Subarachnoid hemorrhage	33	1.37%	1044714	0.20%
Intracerebral hemorrhage	9	0.37%	1066221	0.20%

Prevalence of neurological disorders among both sexes and all ages groups in the InterTAK Registry and in the Global Burden of Disease study. The prevalence of seizure/epilepsy and subarachnoid hemorrhage seems to be higher in the InterTAK Registry than in the Global Burden of Disease statistics, while numbers of ischemic stroke and intracerebral hemorrhage were similar in both cohorts.
*Includes data for countries which are participating in the InterTAK Registry (Australia, Austria, Czech Republic, Finland, France, Germany, Italy, New Zealand, Poland, Russia, United Kingdom, and USA). USA: data for California, Florida, Iowa, Kentucky, and Minnesota were included. UK: data for Greater London, Oxfordshire, and Bournemouth were included.
The numbers used for patients in the general population were extracted from Global Health Data Exchange (GHDx; http://ghdx.healthdata.org/gbd-results-tool) for the year 2019.[1]

Reference:

1. Global Burden of Disease Collaborative Network. Global Burden of Disease Study 2019 (GBD 2019) Results. 2020.

Supplementary Figure 3. Causes of in-hospital death.

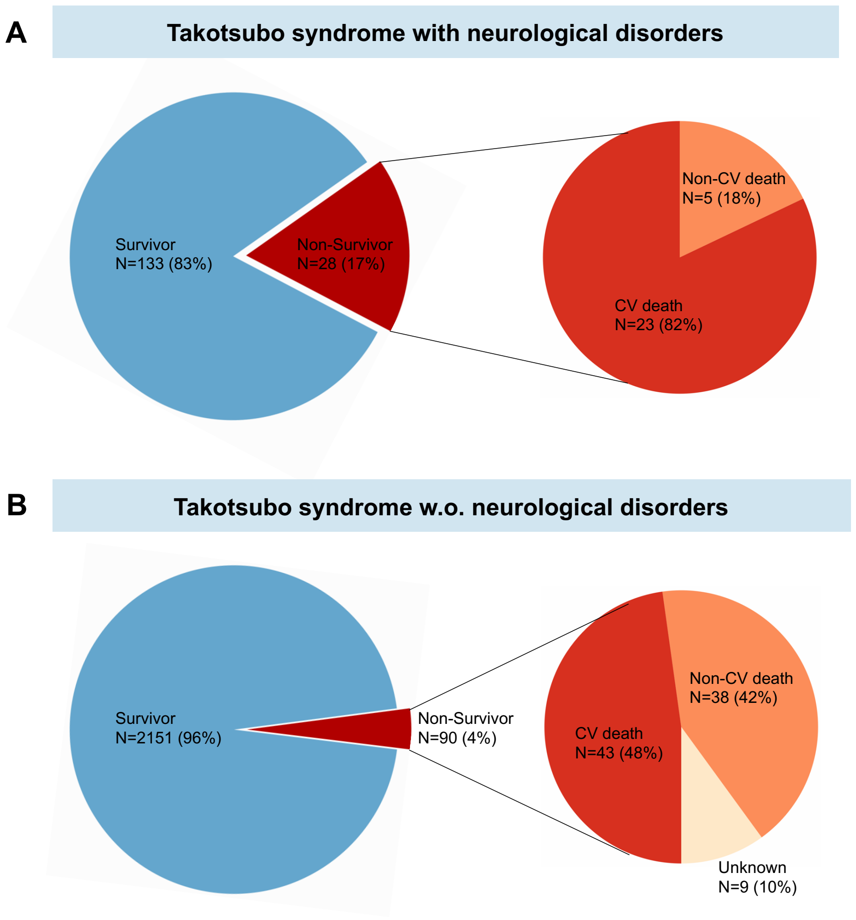

Causes of in-hospital death in TTS patients with primary neurological disorders (A) and in TTS patients without neurological disease (B).
CV death cardiovascular death; non-CV death non-cardiovascular death; TTS takotsubo syndrome.

Supplementary Figure 4. Long-term outcome analysis.

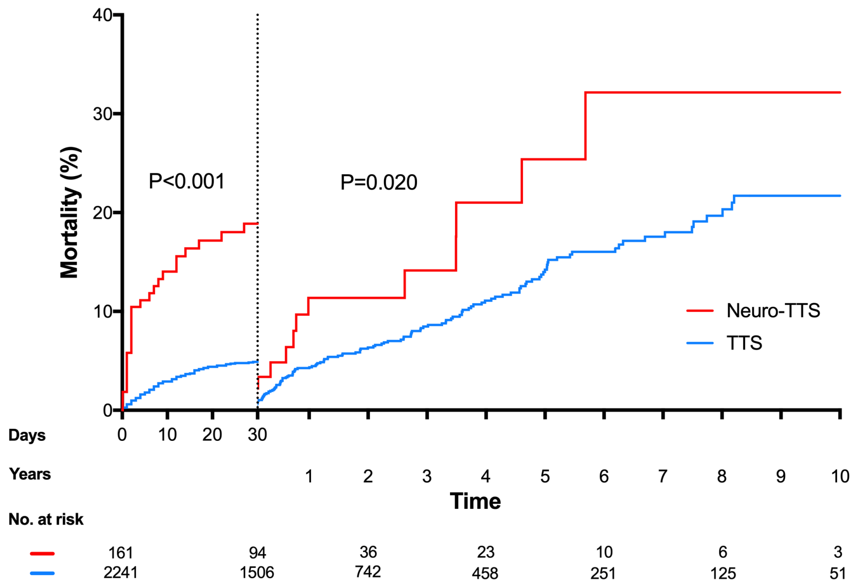

10-year landmark analysis demonstrated increased mortality of TTS patients with neurological disorders within the first 30 days (P<0.001) and after the landmark of 30 days up to 10 years (P=0.020).
TTS takotsubo syndrome.
